# Phenology-dependent variation in the non-structural carbohydrates of broadleaf evergreen species plays an important role in determining tolerance to defoliation (or herbivory)

**DOI:** 10.1038/s41598-017-09757-2

**Published:** 2017-08-31

**Authors:** Zhicheng Chen, Lin Wang, Yongxin Dai, Xianchong Wan, Shirong Liu

**Affiliations:** 1 Institute of New Forestry Technology, Chinese Academy of Forestry, Beijing, 100091 China; 2 College of Forestry, Shanxi Agricultural University, Taigu, 030800 China; 3 Laboratory of Forest Ecology and Environment of State Forestry Administration, Institute of Forest Ecology, Environment and Protection, Chinese Academy of Forestry, Beijing, 100091 China

## Abstract

Two broadleaf evergreen canopy species (*Schima superba* and *Engelhardia roxburghiana*) with different phenologies in a subtropical region of southern China were used to determine the influence of leaf phenology on the impact of an insect pest attack. *S*. *superba* regenerates its leaves in February, while *E*. *roxburghiana* regenerates its leaves in May. The moth *Thalassodes quadraria* attacked the two broadleaf evergreen species in March to April, and the newly produced leaves were removed for *S*. *superba* but not for *E*. *roxburghiana*. The young trees were artificially defoliated to imitate an insect pest attack during March 2014. Nonstructural carbohydrate (NSC) and growth measurements and a retrospective analysis based on the radial growth of mature trees were conducted in January 2015. The results showed that NSC concentrations decreased in *S*. *superba* during canopy rebuilding, and the subsequent defoliation severely inhibited leaf and shoot growth, prevented NSC restoration in roots and stem xylem, and caused high mortality. The insect outbreaks reduced the radial growth of *S*. *superba*. In contrast, *E*. *roxburghiana* experienced less growth retardation, lower mortality, and normal radial growth. Thus, taking phenology-dependent variation in NSCs into consideration, defoliation and insect pest outbreaks more negatively impacted *S*. *superba* than *E*. *roxburghiana*.

## Introduction

In recent years, forest mortality has increased on a global scale. This phenomenon has been attributed to the drought and drought-induced pest attacks associated with climate change^1, 2^. Regardless of the type of stressor involved, amounts of nonstructural carbohydrates (NSCs), which provide energy for plant growth and the maintenance of life, are likely to decrease, thereby disturbing the equilibrium between plant carbon uptake and utilization. This imbalance can result in plant death^3^. The leaf is the photosynthesis apparatus that produces carbohydrates, including NSCs. Defoliation by any means, including insect predation, will decrease plant host carbon uptake and thereby likely reduce its carbon storage. In general, moderate and severe defoliation cause reductions in NSC concentrations^4–6^. In some cases, the fast recovery of NSC stores after defoliation has been reported^7–9^. Nevertheless, the impact of insect herbivore defoliation varies with the host plant type^10^, growth stage^11, 12^ and nitrogen content and expansion rate of young leaves^13, 14^.

Leaf phenology has been shown to influence plant host resistance to insect predation due to relative differences in foliage chemical composition^15^ and toughness^16^. Phenological asynchrony between budburst and the emergence of larvae can function as an effective mechanism by which plants can protect themselves against defoliating insects that are active in springtime, when the leaves newly emerge and are relatively vulnerable^11^. However, to our knowledge, it remains unknown whether phenology-dependent variation in tree NSC concentrations influences the impact of insect pest predation. It is well known that carbon reserves vary among different tree phenological events^17, 18^ and that the lowest NSC concentrations occur during canopy rebuilding, especially in branches^17^. Carbohydrate reserves play a critical role in tree survival under stress situations^19, 20^, including tolerance to biotic agents^3^. Biotic attack may amplify, or be amplified by, carbon starvation^3^. However, declining carbohydrate reserves could also cause hydraulic failure through the impaired refilling of embolized conduits^21, 22^ and be vulnerable to water deficit. Forest decline is frequently ascribed to the combined effects of drought and pest damage^1, 3^.

A typical natural monsoon evergreen broadleaved forest is the climax vegetation in the subtropical region of the Dinghu Mountains located in central Guangdong, southern China. In recent years, insect pest outbreaks have frequently occurred in the Dinghu Mountains^23, 24^. Severe attacks of the moth *Thalassodes quadraria* Guene occurred in this region during 1985–1989^23^ and 2012–2013. After the 2012–2013 infestation, one dominant canopy species, *Schima superba* Gardn. et Champ., suffered high mortality, whereas the mortality of another dominant canopy species, *Engelhardia roxburghiana* Wall., was significantly lower despite the fact that the severity of the insect pest attacks on both tree species was similar. The Dinghu Mountain region has a typical monsoon climate with a distinct dry season from October to March. Insects frequently emerge there in early spring (March to April). Since 1980, the mean air temperatures in southern China have gone up, and rainfall patterns have shifted towards more severe storms during the wet season, more rain-free days during the year and fewer days per year with light rain. Total rainfall, however, has not significantly changed^25^. Both *S*. *superba* and *E*. *roxburghiana* are broadleaved evergreen species. *S*. *superba* regenerates its leaves each February, whereas *E*. *roxburghiana* produces its foliage in May^26^ (Supplemental Fig. 1). Therefore, *S*. *superba* renews its leaves just before the insect outbreak, whilst *E*. *roxburghiana* renews them after insect emergence.

We hypothesize that (1) variation in nonstructural carbohydrates (NSCs) with the phenology cycle play an important role in the differential responses of *S*. *superba* and *E*. *roxburghiana* to insect pest attacks, and (2) the differential responses of the two species to insect pest attacks may also be related to the interaction between carbon source limitation caused by the insect predation and water deficit in the dry season. Canopy rebuilding consumes much of the carbohydrate reserves, and if the new leaves are immediately defoliated by pests, the plants could face carbon starvation. However, the declining carbohydrate reserves could also cause hydraulic failure through the impaired refilling of embolized conduits in the dry season. To test these hypotheses, we carried out an artificial defoliation experiment simulating an insect attack. Before and after the defoliation of young trees, the nonstructural carbohydrate content in different tissues was measured. The growth rates, gas exchange, and water relations were analysed after defoliation. In addition, a retrospective analysis based on the ring growth of mature trees was conducted to measure radial growth responses to the insect outbreaks. Tree ring analysis has proven to be a suitable tool for measuring growth reduction, and it serves as an indicator of previous environmental stress and insect attacks^27^.

## Results

### Impact of defoliation on growth

**Figure 1:**
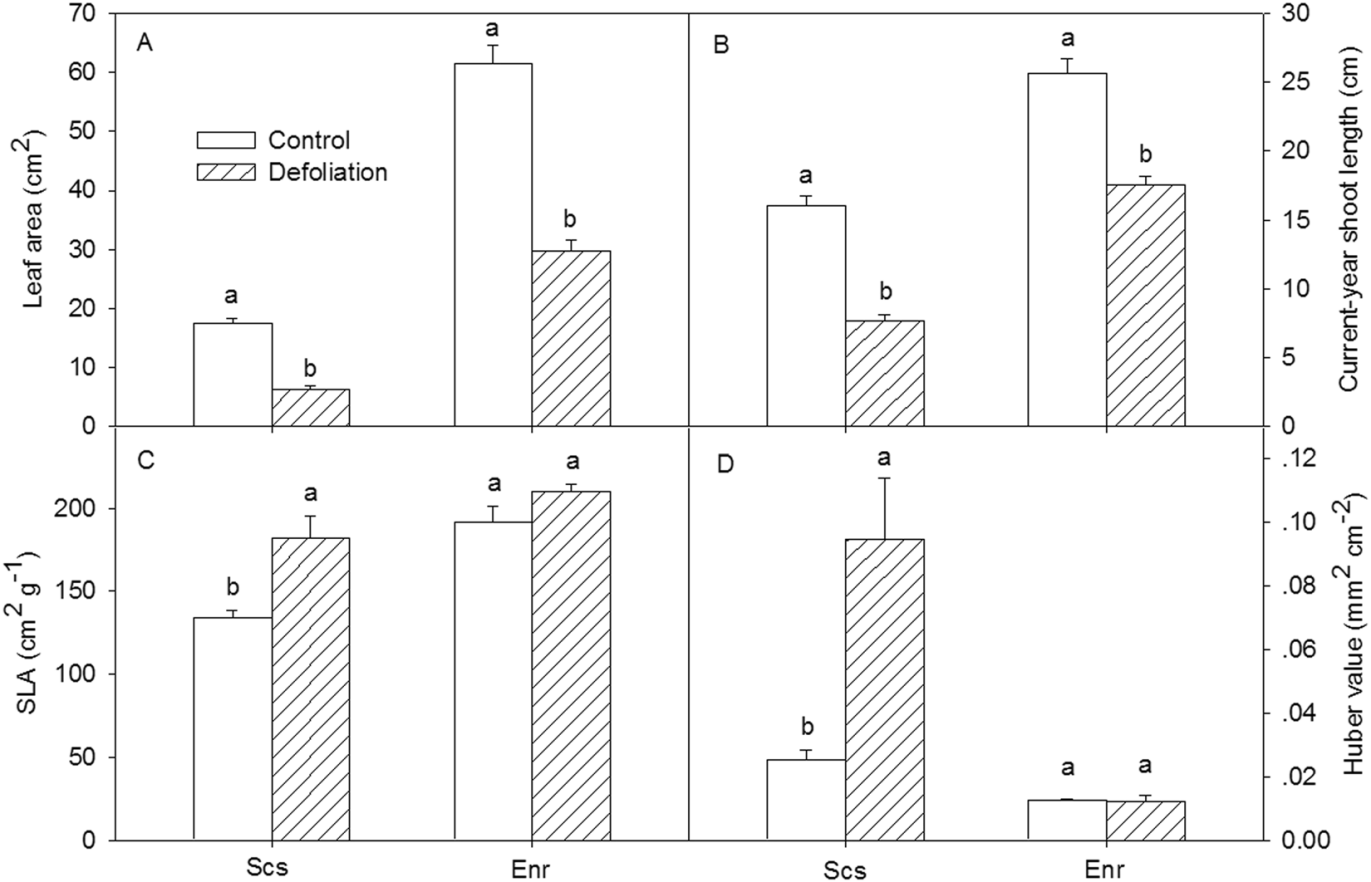
(**A**) Leaf size, (**B**) current-year shoot length, (**C**) specific leaf area, and (**D**) Huber value for control and defoliated *Schima superba* (Scs) and *Engelhardia roxburghiana* (Enr). The mean ± SE is presented (*n* = 3–6), and different letters above the bars indicate significant differences in the values at P = 0.05.

**Figure 2:**
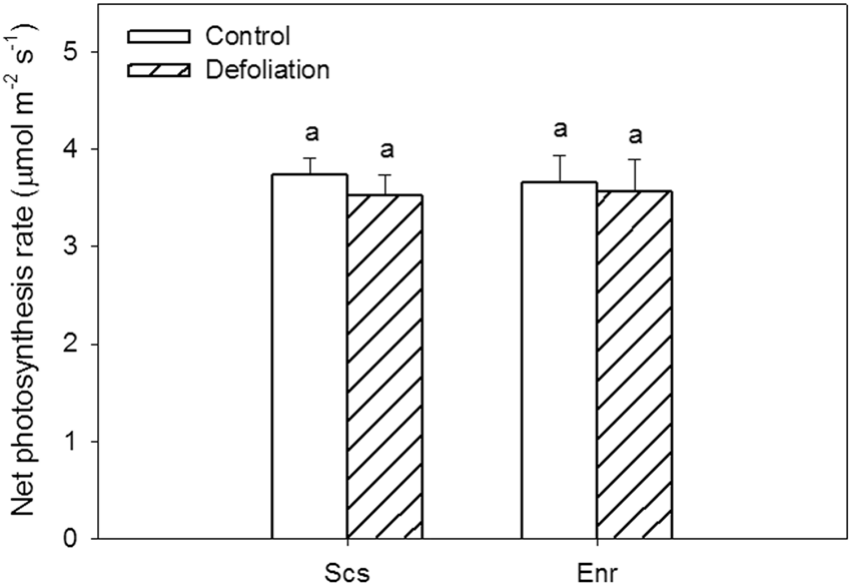
Net photosynthetic rates of *Schima superba* (Scs) and *Engelhardia roxburghiana* (Enr). The mean ± SE is presented (*n* = 3–6), and different letters indicate a significant difference in the net photosynthetic rates at P = 0.05.

Defoliation significantly reduced newly grown leaf size and current-year shoot length in both species (Fig. 1A,B). The leaf size and shoot length of defoliated *S*. *superba* were reduced by 64.2% and 52% in comparison to the control (p < 0.01), respectively, while those of the defoliated *E*. *roxburghiana* decreased by 51.7% and 31.7% in comparison to the control (p < 0.01), respectively. There were significant interactions between species and treatment (p < 0.01). These findings indicate that the adverse effects of defoliation on the leaf size and shoot length were more severe in *S*. *superba* than in *E*. *roxburghiana*. Defoliation significantly increased both the specific leaf area (SLA) and the Huber value (Hv) of *S*. *superba* (p < 0.01) but did not change those of *E*. *roxburghiana* (p = 0.67) (Fig. 1C,D). The Hv of defoliated *S*. *superba* was 3.72 times greater than that of the control. The SLA and Hv data indicate that the leaf dry matter and the total leaf area per unit sapwood area of defoliated *S*. *superba* were still lower than those of the control even ten months after defoliation. Nevertheless, there were no significant differences (p > 0.60) between the defoliation treatment and the control for either tree species in terms of net photosynthetic rate (P_n_) per unit leaf area (Fig. 2). However, the total assimilation per tree was much lower in the defoliated *S*. *superba* than in the control because the total leaf area of the defoliated treatment was significantly reduced compared to that of the control.

### Impact of defoliation on survival

Three of the six defoliated young *S*. *superba* trees died off within ten months, whereas all six defoliated young *E*. *roxburghiana* trees survived. All of the control trees from both species survived.

### Impact of defoliation on NSCs in different tissues

**Figure 3:**
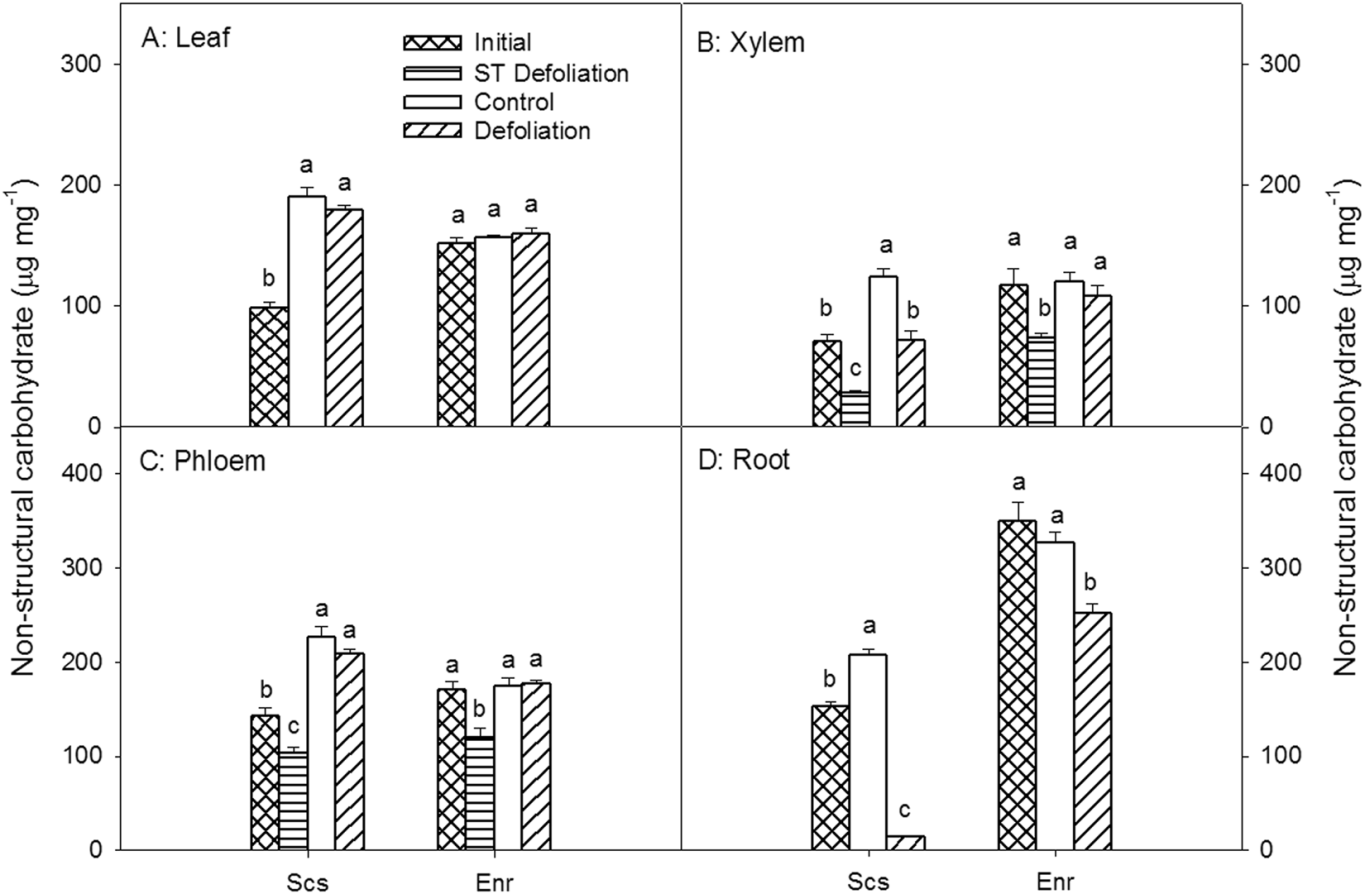
Nonstructural carbohydrate (NSC) concentrations in leaves, xylem, phloem, and roots of *Schima superba* (Scs) and *Engelhardia roxburghiana* (Enr). The sum of SS and starch is considered as the NSCs. Samples denoted Initial were collected in March 2014, and samples denoted Control and Defoliation were collected in January 2015. The mean ± SE are presented (*n* = 3–6), and different letters indicate significant differences in NSCs in different tissues at P = 0.05.

The total nonstructural carbohydrate (NSC) concentrations in the *S*. *superba* leaves, xylem, phloem and roots sampled in March 2014 (when leaf renewal was completed and just before the defoliation treatment) were all significantly lower than those of the control samples collected in January 2015 (p < 0.01), prior to the next cycle of leaf renewal. There were no significant differences in NSC concentrations among *E*. *roxburghiana* organs and tissues collected between March 2014 and January 2015 (p > 0.85) (Fig. 3). The defoliated *S*. *superba* had significantly lower NSC concentrations in xylem and roots than the control (p < 0.01); the NSC concentrations in the roots of the defoliated *S*. *superba* were only 6.6% that of the control. There were no significant differences in leaf (p = 0.96) and stem phloem (p = 0.88) NSC concentrations between defoliated and control *S*. *superba*. The NSC concentrations of leaves, xylem and phloem from the defoliated *E*. *roxburghiana* were not significantly different from those of the control (p > 0.95). Only the NSC concentration in the roots of defoliated *E*. *roxburghiana* was significantly lower than that of the control (p = 0.024). However, the absolute NSC concentration in the roots of defoliated *E*. *roxburghiana* was much higher than that of defoliated *S*. *superba* (p < 0.01).

**Figure 4:**
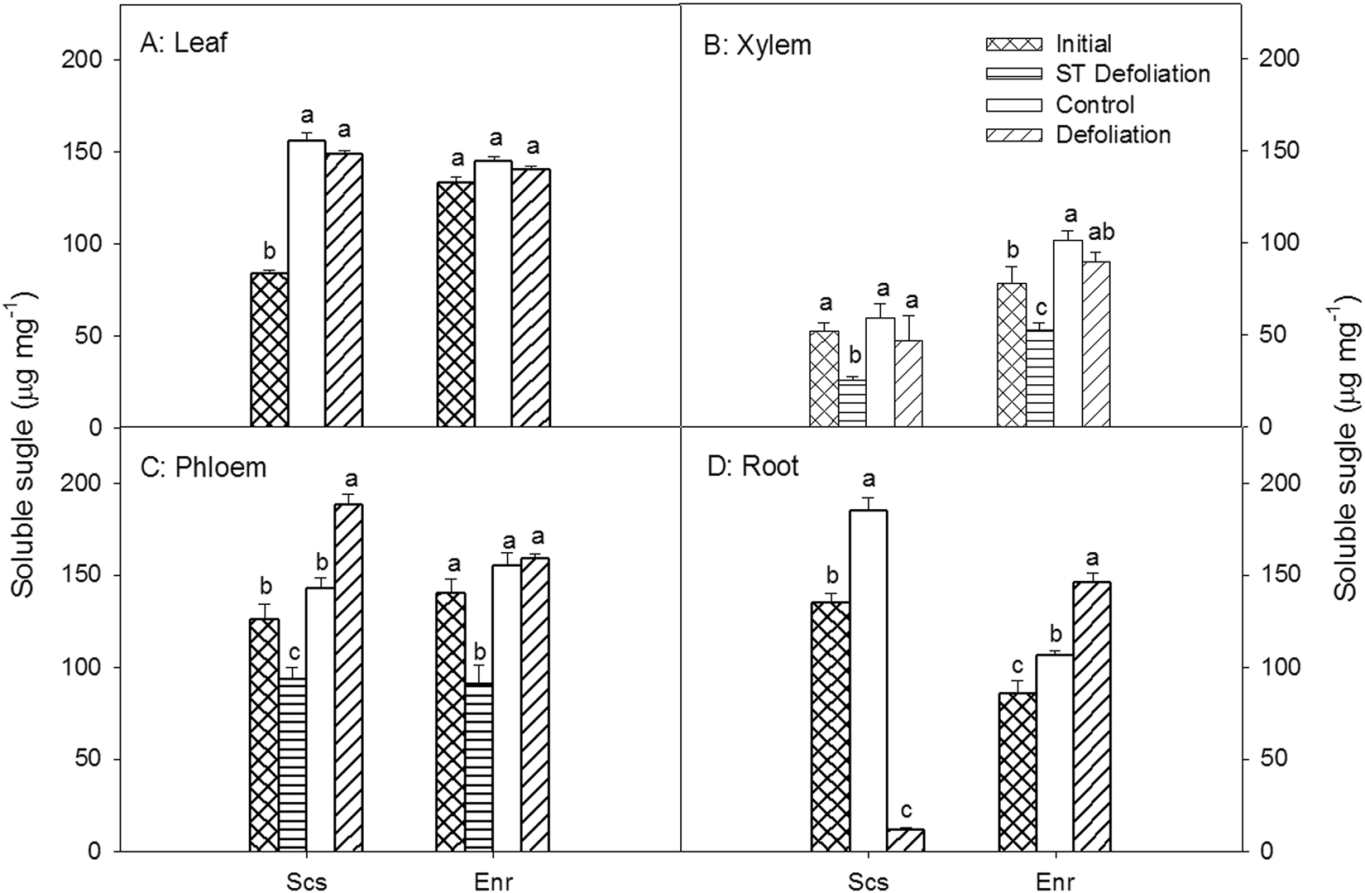
Soluble sugar (SS) concentrations in leaves, xylem, phloem, and roots of *Schima superba* (Scs) and *Engelhardia roxburghiana* (Enr). Samples denoted Initial were collected in March 2014, ST Defoliation samples were collected in April 2014, 20 days after defoliation, and samples denoted Control and Defoliation were collected in January 2015. The mean ± SE is presented (*n* = 3–6), and different letters indicate significant differences in SS in different tissues at P = 0.05.

In the samples tested, the NSCs consisted mainly of soluble sugars (SSs) except in *E*. *roxburghiana* roots, where starch (St; data not presented) accounted for a slightly greater portion of the NSCs than did SS (Figs 3 and 4). The SS concentrations in the *S*. *superba* leaves and roots sampled in March 2014 were all significantly lower than those of the control samples collected in January 2015 (p < 0.01), while no significant differences in the xylem and phloem SS concentrations of the samples collected at the different times were observed (p > 0.37). There were no significant differences in SS concentrations among *E*. *roxburghiana* organs and tissues collected between March 2014 and January 2015 (p > 0.85). In both species, the SSs in all defoliated tissues except for the *S*. *superba* roots were not lower than those of the corresponding tissues in the controls.

The NSCs and SSs of defoliated shoots sampled in April, just 20 days after defoliation, were also measured. Consistently, the SS and NSC concentrations in the xylem and phloem of the defoliated shoots sampled in April in both species were all significantly lower than those measured at any other time. The NSC concentrations in the xylem and phloem and the SS concentration in the xylem of *S*. *superba* were lower than those in the corresponding tissues of *E*. *roxburghiana*, suggesting that defoliated *S*. *superba* that had just finished renewing leaves immediately mobilized the remaining NSCs over the short term for resprouting, resulting in the lower NSC reserve in *S*. *superba*.

### Impact of defoliation on hydraulic status

**Figure 5:**
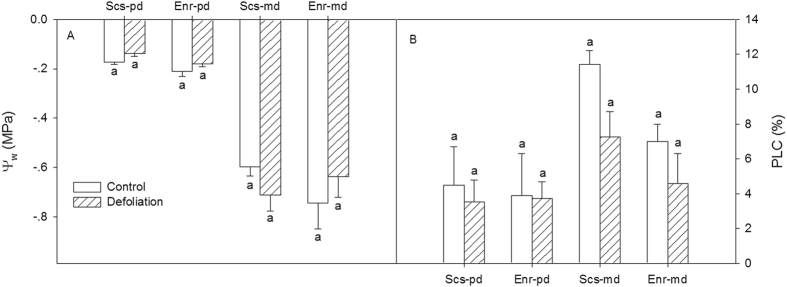
(**A**) Predawn (pd) and midday (md) water potential, and (**B**) predawn and midday PLC (percentage loss of hydraulic conductivity) for the control and defoliation treatment for *Schima superba* (Scs) and *Engelhardia roxburghiana* (Enr). The mean ± SE is presented (*n* = 3–6), and different letters indicate significant differences in the values at P = 0.05.

**Figure 6:**
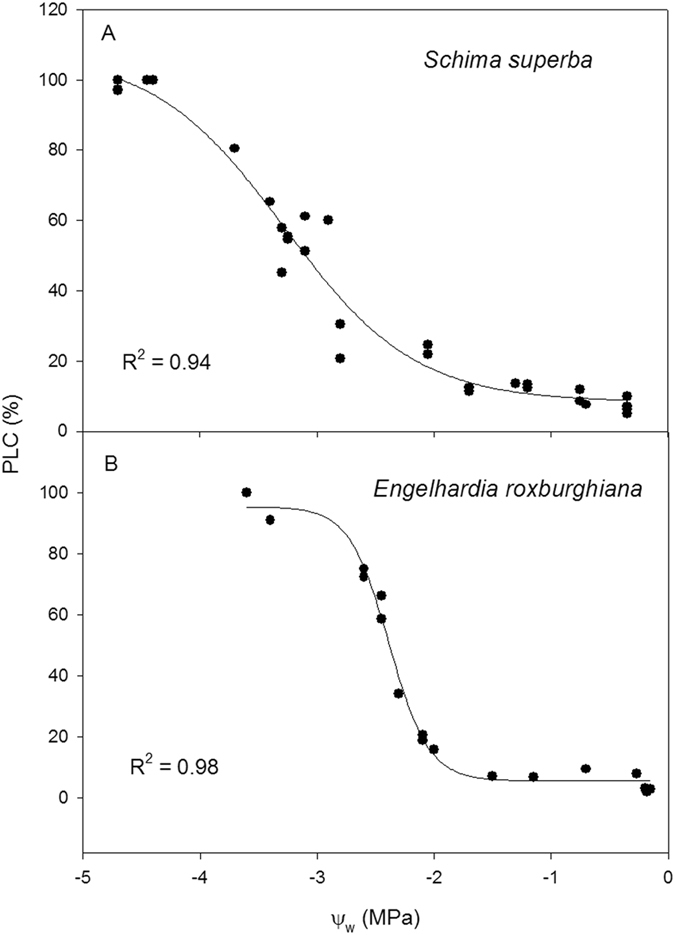
Xylem vulnerability curves to cavitation for *Schima superba* (Scs) and *Engelhardia roxburghiana* (Enr).

Defoliation had no significant effect on predawn water potential, midday water potential, predawn PLC or midday PLC in either species (Fig. 5). For both the control and the defoliation treatment, the predawn potentials of the two species were high, at approximately −0.1~−0.2 MPa, and their midday water potentials were not lower than −0.8 MPa. The predawn and midday PLC values were relatively small, which indicates that the hydraulic status of the two species was normal and that hydraulic failure did not occur under the local weather conditions. P_50_ is the water potential at which 50% of the hydraulic conductivity is lost, and it is an indicator of the resistance of xylem to air embolism. The P_50_ of *E*. *roxburghiana* was −2.2 MPa, while that of *S*. *superba* was −2.8 MPa (Fig. 6); these values, together with the xylem vulnerability curves (VC), showed that the former was more susceptible to cavitation than the latter.

### Tree ring analysis of the two species

**Figure 7:**
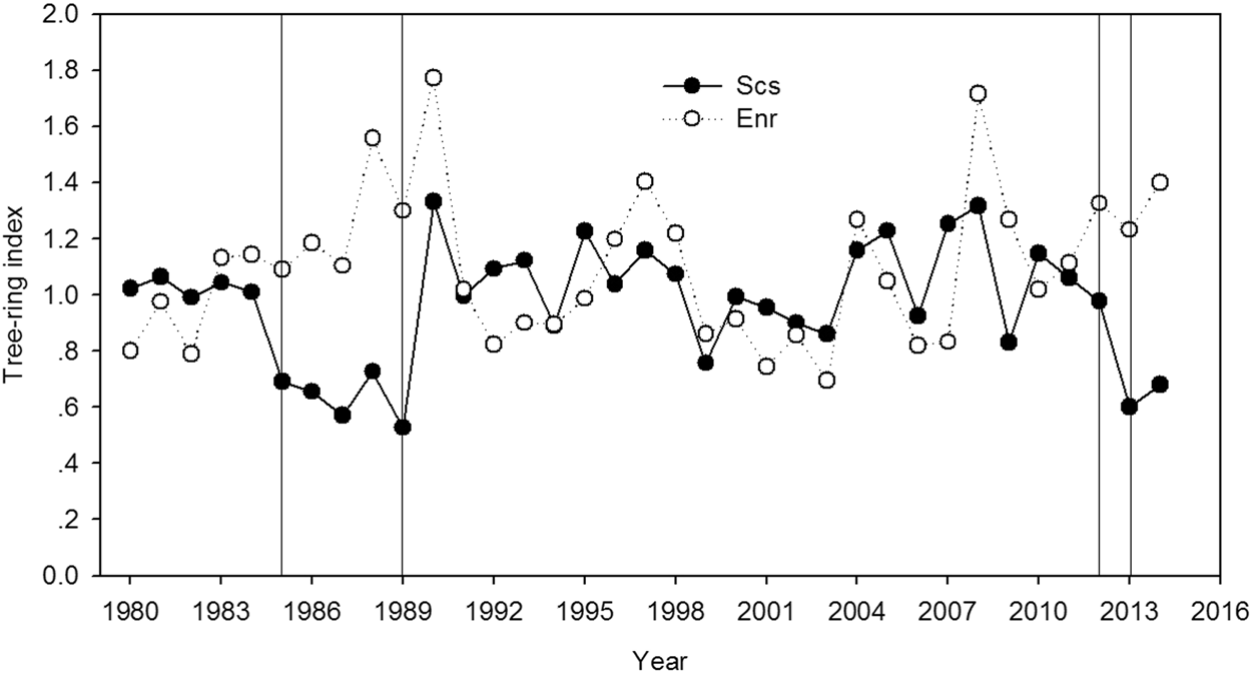
Tree ring indices for *Schima superba* (Scs) and *Engelhardia roxburghiana* (Enr) for the period of 1980–2014. The areas between two vertical lines indicate the insect outbreak periods.

A retrospective analysis based on ring growth showed that the growth trend of *S*. *superba* was lower than that of *E*. *roxburghiana* during the insect pest outbreak periods (1985–1989 and 2012–2013), as shown in the tree ring chronologies (Fig. 7).

## Discussion

The concentrations of NSCs in a tree are indicative of the balance between the carbon being fixed and that being utilized for growth and metabolism or exuded below ground^18^. Carbon reserves fluctuate along with plant phenological events^17, 18^. The impacts of the loss of carbon reserves in the leaves on trees depend upon the time of year in which the leaves are lost^28–30^. In the studied region, all of the insect outbreaks occurred in March–April, just after *S*. *superba* renews its leaves (February). In contrast, *E*. *roxburghiana* renews its leaves in May, after the insect outbreaks^26^. The observed data in this study showed that the *S*. *superba* NSC concentrations in March (when *S*. *superba* had just produced new leaves) were significantly lower than those found in January (before annual leaf renewal, which was considered as the control). These observations suggest that the leaf renewal process consumed much of the NSC reserves in all of the tissues tested. However, there were no significant differences in NSC concentrations for any of the *E*. *roxburghiana* tissues sampled from control (non-defoliated) trees between March 2014 and January 2015. As stated above, the annual leaf renewal of *E*. *roxburghiana* occurs in May; therefore, the NSC concentrations in the samples collected in March 2014 and in January 2015 might not be affected by leaf renewal. The results in this study exhibited that carbon reserves in the two species fluctuated along with their respective phenological events. In artificial defoliation imitating an insect pest attack during March, the leaves that were newly produced were removed for *S*. *superba*, but the leaves that were almost one-year-old were removed for *E*. *roxburghiana*. As a result, during canopy rebuilding, the NSC concentrations decreased in *S*. *superba*, and the subsequent defoliation severely prevented NSC restoration in the roots and the stem xylem. In contrast, NSCs were restored in *E*. *roxburghiana* to much closer to the control levels, even in roots.

The storage of nonstructural carbon is of vital importance to woody plants because the NSC reserves enable long-lived organisms to tolerate biotic and abiotic stress, including pests, physical disturbances and drought^31, 32^. The carbon starvation hypothesis indicates that the balance between plant carbon uptake and expense is upset during long-term external stress, resulting in reduced growth and even death^3, 21, 33, 34^. In this study, artificial defoliation simulating insect pest attack was conducted in March 2014. In January 2015, following an entire growing season, growth rates and physiological parameters, including NSC concentration, were measured. With the NSC reserves of *S*. *superba* already reduced by leaf renewal, the subsequent defoliation significantly decreased the NSC concentrations in the roots and xylem even further—especially in the roots, where the NSC concentration was reduced to 6.6% of the control level. In contrast, the NSC concentration of the defoliated *E*. *roxburghiana* remained at 77.2% of the control level.

Long-term or severe suppression of carbon fixation can lead to a significant reduction in root carbon reserves^35^. It has been indicated that the carbohydrate reserves in the roots of defoliated trees remained low for up to two growing seasons, which is consistent with the fact that we found that the root NSC concentrations were still low even 10 months after defoliation, particularly in the case of *S*. *superba*. The carbon reserves of tree roots play a critical role in survival and the restoration of growth rates after defoliation^19, 20, 36^. The low survival rates of *S*. *superba* post-defoliation may be ascribed to its extremely low root NSC reserves. The sink hierarchy hypothesis proposes that the re-establishment of root NSC reserves after defoliation is hindered by the fact that the root system is at the end of the carbon transport chain and therefore has a lower priority for carbohydrate allocation despite the fact that it is a large carbon sink^37^. There were significant differences between the two tree species in terms of post-defoliation root NSC concentrations. This fact may account for the different responses of the two host species to the insect pest attacks in recent years.

It is well known that defoliation significantly lowers the rates of tree growth, including growth in woody tissues^38, 39^ and branch size^36^. In this study, leaf size and new shoot length in both *S*. *superba* and *E*. *roxburghiana* decreased in response to defoliation. Such changes have also been reported in previous studies^22, 38^. Taking interactions with the phenology cycle into consideration, defoliation impeded the growth of *S*. *superba* more than that of *E*. *roxburghiana*. The Huber value of *S*. *superba* shown in Fig. 1 significantly increased, suggesting a decrease in the total leaf area (Supplemental Fig. 2). Because leaves constitute the photosynthetic apparatus, they directly determine post-defoliation carbon recovery^40^. The increased SLA of defoliated *S*. *superba* (relative to the control) indicated that the amount of dry matter per leaf area decreased, which is commonly observed after defoliation^38^. The net photosynthetic rates per unit area in both species subjected to defoliation were restored to the corresponding control levels. The total assimilation capacity would therefore be mainly determined by the total leaf area (Supplemental Fig. 2). The lower total leaf area in defoliated *S*. *superba* may have limited its restoration of NSC and its growth. The reductions in the radial growth of mature *S*. *superba* trees during the pest outbreaks and the subsequent years also provided evidence that both assimilation and growth rates were reduced in infested *S*. *superba* trees.

The NSC concentrations of the aerial parts, including the *E*. *roxburghiana* leaves, xylem and phloem and the *S*. *superba* phloem, were restored to control levels within ten months after defoliation, while the root NSC reserves were not. These findings lend credence to the statement that new photosynthate is distributed preferentially to the shoots and leaves rather than the roots^30, 36, 41, 42^. This distribution pattern of new photosynthate favours the growth of the photosynthesis apparatus required for carbon fixation. Unlike in *E*. *roxburghiana*, the NSC concentration in the xylem of defoliated *S*. *superba* was not restored to the control level. This observation provided additional evidence that *E*. *roxburghiana* restored its NSC reserves more effectively than did *S*. *superba*.

The NSC concentrations of the aerial tissues were restored to levels of or close to those of the control. However, the total leaf area and the current-year shoot length were significantly lower than those of the control, especially for *S*. *superba*. This discrepancy suggests that NSC restoration was more successful in the recovery process than was growth re-establishment. This finding aligns with those reported in previous studies^38, 43, 44^. To improve their chances of survival when facing external stressors, trees may inhibit their growth activity^21, 45^. Alternatively, direct constraints on growth (sink limitation) could also lead to increased NSC availability for storage^9, 46^. The relatively fast recovery of NSC reserves in the absence of growth may also be related to greater NSC availability in the absence of the strong sink strength of growing buds^46^. Additionally, the reduced growth with a decrease in leaf area may not necessarily be due to carbon limitation but to other factors, such as hormonal control after a reduction in the sink limitation from leaves.

Regardless of the treatment, the predawn water potentials for both *S*. *superba* and *E*. *roxburghiana* were relatively high (−0.13~−0.2 MPa). The midday water potentials were not lower than −0.8 MPa. The mean midday PLC did not exceed 12%. The maximum PLC was less than 20%. Our results were similar to those reported in a recent study conducted in the same geographic region as ours, in which a mean predawn water potential of −0.31 MPa was found for forty-eight indigenous tree species during the mid-dry season^47^. This finding indicates that the potential risk of hydraulic failure due to seasonal drought was low. Furthermore, the P_50_ of *S*. *superba* was −2.8 MPa, while the P_50_ of *E*. *roxburghiana* was −2.2 MPa, indicating that the xylem resistance to cavitation of *S*. *superba* was higher than that of *E*. *roxburghiana*. The aforementioned results and information may not support the hypothesis that the combination of seasonal drought and carbon starvation was more detrimental to *S*. *superba* than to *E*. *roxburghiana*. The seasonal drought in the experimental region was relatively mild by then and might not contribute to reduced growth and increased mortality after defoliation. In addition, the young trees all grew in the understory, and the transpiration demand was likely low. Thus, the morphological and physiological differences between the two species after defoliation may be due mainly to the different degrees of carbon deficit.

The annual and seasonal temperatures in both the dry and wet seasons in the Dinghu Mountains region have significantly increased since 1954. In addition, the annual number of rain-free days has significantly increased since 1980^25^. The information reported above suggests that climate change trends seem to promote *T*. *quadraria* outbreaks, and in general, the emergence of *T*. *quadraria* tends to occur in warm humid regions, such as the Dinghu Mountains^23^. With global climate change and the differential responses of the two species to *T*. *quadraria* attacks, *S*. *superba* is expected to decline in the forest over the longer term. No such insect outbreaks are known to occur at higher elevations or in colder regions.

## Conclusions

Our results indicate that during the canopy rebuilding phase of the tree phenology cycle, the NSC concentrations decreased in *Schima superba* leaf, stem xylem, stem phloem and root tissues. Artificial defoliation that simulated an insect pest attack severely inhibited both leaf and shoot growth and prevented the restoration of NSCs in the storage tissues of *S*. *superba* roots and xylem after ten months. These effects led to high tree mortality rates. A retrospective analysis based on ring growth rates in mature trees showed that outbreaks of the insect *T*. *quadraria* reduced radial growth in *S*. *superba*. In contrast, another canopy species, *Engelhardia roxburghiana*, has a different phenology cycle from that of *S*. *superba*. *E*. *roxburghiana*, which renews its leaves (in May) after the insect outbreak (in March-April), suffered far fewer adverse effects of defoliation and insect pest attacks than did *S*. *superba*. Although defoliation reduced leaf and shoot growth rates in *E*. *roxburghiana* as well, the extent of the reduction was less than that seen in *S*. *superba*. The NSCs in the defoliated *E*. *roxburghiana* recovered almost to control levels within ten months after defoliation. Tree ring analysis indicated that *E*. *roxburghiana* radial growth is not impeded by the insect pest outbreaks. Thus, when their interactions with phenology are taken into account, defoliation and insect pest outbreaks hindered the growth rates, NSC restoration and survival rates of *S*. *superba* more than those of *E*. *roxburghiana*. The water relation measurements in this study did not demonstrate any interaction between source limitation and water deficit.

## Methods

### Study site and experimental design

This study was conducted at the Dinghu Mountains Forest Ecosystem Research Station (DFERS; 23°09′21″–23°11′30″N, 112°32′39″–112°35′41″E), Chinese Academy of Sciences, central Guangdong, southern China. The region is characterized by a typical low subtropical monsoon humid climate, with a dry season from October to March. The mean annual total precipitation is approximately 1900 mm, of which nearly 80% occurs during the wet season (April to September). The mean annual temperature is 21.4 °C, with the lowest and highest monthly mean temperatures of 12.6 °C in January and 28.0 °C in July, respectively. The mean relative humidity is 77.7%. The bedrock includes sandstone and shale. The soils are ultisols and udults with a pH of 4.0–4.9. There are three major forest types in this region: pine (early successional stage), mixed pine and broadleaf (mid-successional stage) and evergreen broadleaf (advanced successional stage).

The objective of this study was to understand why the two evergreen broadleaf species in this region, namely, *Engelhardia roxburghiana* Wall. (Juglandaceae) and *Schima superba* Gardn. et Champ. (Theaceae), responded differently to insect pest attacks in recent years^23^. These two trees are canopy species in the DFERS. Insect [*T*. *quadraria* (Geometridae of Lepidoptera)] outbreaks occurred periodically in recent years in the DFERS region. The latest occurrences were in March and April of 2012 and 2013. The extent of insect infestation was similar for both *E*. *roxburghiana* and *S*. *superba*, with defoliation of more than 70% based on a field survey we conducted in late April of 2013 and consultation with the forest ranger in the national reserve. We selected twelve young trees each of *S*. *superba* and *E*. *roxburghiana*, all similar in size and growing naturally in the Dinghu Mountains. The young trees were all naturally regenerated and grew in the understory of the evergreen broadleaf forest. The average diameter at breast height (DBH) and height of *S*. *superba* were 1.84 (±0.57, ±SE) cm and 2.77 (±0.97) m, respectively, and for *E*. *roxburghiana*, the average measurements were 1.28 (±0.33) cm and 2.76 (±0.88) m, respectively. Six young trees per species were used as test subjects for simulating insect attacks in terms of leaf chewing, whilst the other six served as controls. We manually removed all of the foliage in March 2014. By January 2015, all six defoliated young *E*. *roxburghiana* trees had survived, but three of the young defoliated *S*. *superba* trees had died. In January 2015, physiological indices of all surviving defoliated young trees and controls were measured. To understand the consequences of carbon stress, including its interaction with seasonal drought, we planned to take the main measurements in the dry season. Additionally, carbon stress may play a role in longer-term feedbacks and pathways to mortality. For the retrospective analysis based on ring growth measurements, twenty trees per species in the same area with DBH values of approximately 30–50 cm and ages of approximately 50 years were studied.

### Growth responses

One sun-exposed twig per tree was collected for growth analyses in January 2015. All of the leaves on each twig were removed and collected for the measurement of the total leaf area with a leaf area meter (Li-3000C; Li-Cor Inc., Lincoln, NE, USA). For each twig, the Huber value (Hv) was calculated as the sapwood area divided by the total leaf area of the cut twig. More than six fully expanded leaves per individual were selected for the determination of the average leaf area (LA). The leaves were then heated at 105 °C for 20 min to stop all enzymatic activity and then oven-dried at 75 °C for 48 h to determine the dry leaf mass. The specific leaf area (SLA) was calculated as the leaf area divided by the dry leaf mass. At least three current-year shoots per tree were measured with a ruler in order to obtain the current-year shoot length. The net photosynthetic rate (P_n_) was measured with a Li-6400XT portable photosynthesis system (Li-Cor Inc., Lincoln, NE, USA) on sunny days in January 2015. The measurements were made between 09:00 h and 11:30 h. Three sun-exposed leaves per tree were selected for the photosynthetic measurements. Measurements were taken using a standard 2 cm × 3 cm chamber equipped with blue-red light emitting diodes (LED) providing a photosynthetic photon flux density (PPFD) of 1500 µmol m^−2^ s^−1^. The ambient CO_2_ concentration and leaf temperature were maintained at 400 ppm and 18 °C during the experiment, respectively.

### Chemical analyses

The leaf, stem and root samples used for the measurement of soluble sugar (SS) and starch (St) were collected in March 2014 (before defoliation) and in January 2015 (ten months after defoliation). The stems were separated into xylem and phloem. All of the tissue samples were heated at 105 °C for 20 min to stop all enzymatic activity and then oven-dried at 75 °C for 48 h. The dried leaves, stem phloem and xylem and fine roots (0.5–5 mm in diameter) were ground and sieved through a 100-mesh screen, and the powder was used for the determination of soluble sugar (SS) and starch (St) content using the anthrone-sulfuric acid method^48^. The SS was first extracted three times from the ground samples using 80% ethanol at 80 °C. The residue obtained after extraction was analysed for starch content by digestion using perchloric acid solution, and the glucose was detected using colourimetry. Following extraction, the concentrations of SS and St were determined photometrically in the presence of anthrone-sulfuric acid reagent using a 96-well microplate photometer (Model SpectraMax 190; Molecular Devices Co., San Francisco, CA, USA) with anthrone-sulfuric acid reagent. The sum of SS and St is considered as the total nonstructural carbohydrates (NSCs).

### Predawn and midday water potential and percentage loss of hydraulic conductivity

Predawn and midday twig water potentials were measured using a portable pressure chamber (Model No. 1000; PMS Instruments Co., Corvallis, OR, USA.) on sunny days in January 2015. The predawn water potential (Ψ_pd_) was measured between 05:00 h and 06:00 h, whilst the midday water potential (Ψ_md_) was measured between 11:30 h and 13:30 h. Two twigs per tree were selected and immediately measured *in situ*.

The maximum xylem vessel length for each species was measured before the percentage loss of hydraulic conductivity (PLC) determination in order to avoid the introduction of errors when cutting branches for the PLC measurements. The maximum vessel length was measured by the “injection air method” as described by Cohen *et al*.^49^ and Wang *et al*.^50^. Long shoots were cut and injected with compressed air (at approximately 0.15 MPa) from the cut end; the other end was immersed in water. Stem segments (1 cm long) were sequentially excised back until bubbling was observed. The length of the remaining stem was identified as the maximum vessel length. The mean maximum vessel lengths for *S*. *superba* and *E*. *roxburghiana* were 27.9 ± 0.5 cm and 38.3 ± 0.6 cm, respectively. Thus, it was ensured that the shoots collected for the PLC measurements were longer than the maximum vessel length.

Samples for predawn and midday PLC measurements were collected at the same time as those used for the determination of twig water potential. Cutting branches in air readily causes inaccurate PLC values^51^. To avoid this problem, a plastic funnel was wrapped around the branch, sealed with tape and filled with distilled water. The branch was then cut under water using sharp pruning shears, and the cut base was immediately transferred to a bucket filled with distilled water. The cut ends of successive segments were submerged under water^51–53^. The leafy end of the branch was then covered with a large black plastic bag. The bucket containing the branches immersed in water was transferred to the laboratory.

The PLC of predawn and midday branches was measured with a low pressure flow meter (LPFM) as described by Sperry *et al*.^54^. The LPFM apparatus was cleaned prior to the PLC measurements by filling it with bleach solution, setting it aside for at least 5 h and then rinsing it with tap water. Stem segments approximately 5 cm in length and 4 mm in diameter were excised under water and then mounted onto the LPFM system, which had been prefilled with ultra-pure, degassed 25 mM KCl solution passed through a 0.22-μm filter. The initial hydraulic conductance (K_i_) was measured gravimetrically by determining the flow rate of potassium chloride (KCl) solution at a pressure differential of 4 kPa. The stem segment was then flushed at a pressure of 1.75 MPa for 1 min in order to remove air embolisms. The hydraulic conductivity was then determined once again at a pressure differential of 4 kPa and set as the maximum hydraulic conductivity (K_max_). The PLC was calculated as follows:

1 $${\rm{PLC}}=100\times ({{\rm{K}}}_{{\rm{\max }}}-{{\rm{K}}}_{{\rm{i}}})/{{\rm{K}}}_{{\rm{\max }}}$$

### Xylem vulnerability curves

Xylem vulnerability curves (VC) were constructed by plotting stem PLC against corresponding xylem tension values obtained via bench dehydration^52, 54^. The branches used in this assay were similar to those used in the predawn PLC measurements except they were longer, forked and collected from naturally growing trees. The branches were removed from the water and dehydrated on the laboratory bench at ambient irradiance and temperature in order to obtain a series of xylem tensions over a range of PLC values. The branches were carefully wrapped in aluminium foil once they reached the desired tension. After one hour of equilibration, a short leafy shoot was cut in air from one fork of each branch at least one maximum vessel length distant from the sample segment used for PLC measurement. The purpose of this procedure was to determine the xylem tension within the pressure chamber. Soon after excising the short shoot, two to four segments used for PLC determination were cut under water and then measured as described above. To acquire enough data to construct complete VC, more than 30 branches were measured for each species. The VCs were fitted using the equation below^55^:

2 $${\rm{PLC}}=100/(1+\exp ({\rm{a}}({\rm{\psi }}-{\rm{b}})))$$

### Ring width measurement

Trees were sampled in the area where frequent insect pest outbreaks have occurred in recent years. One core from the south side of each tree was extracted at breast height using 5-mm diameter increment borers. In total, 20 cores were collected for each species. The samples were taken to the laboratory, dried, mounted and sanded. Tree ring widths were measured to the nearest 0.01 mm using a LINTAB system (Model No. 6; Rinntech Instruments Co., Heidelberg, Germany). The cross-dating control was evaluated using the COFECHA program^56^. The chronologies were constructed using the ARSTAN program^57^. Age- and growth-related trends in tree ring series were fitted to a negative exponential curve.

### Data analysis

The data were log transformed to meet the assumptions of homogeneity of variance and normality when necessary. We examined the effect of defoliation on carbohydrate reserves in each tissue via repeated-measures ANOVA (over time; control versus defoliation). The other data were analysed using two-way ANOVA (species and treatments) followed by Student’s *t* multiple comparisons. All analyses were performed using SAS v10 software (SAS Institute Inc., Cary, NC, USA). All statistically significant differences were tested at α = 0.05. Only three defoliated young *S*. *superba* trees were surviving in January 2015; therefore, only three replicates were available for this particular treatment. All other species and treatments had six replicates.

## Electronic supplementary material

Figures
