# Supplementary material for: Phenology-dependent variation in the non-structural carbohydrates of broadleaf evergreen species plays an important role in determining tolerance to defoliation (or herbivory)
Source: Sci Rep. 2017 Aug 31;7:10125. doi: 10.1038/s41598-017-09757-2 (PMC5579018; doi:10.1038/s41598-017-09757-2)
Supplement: Supplementary file 1 — Figures [file 41598_2017_9757_MOESM1_ESM.doc]

**Phenology-dependent variation in the non-structural carbohydrates of broadleaf evergreen species plays an important role in determining tolerance to defoliation (or herbivory)**

Zhicheng Chen^1^, Lin Wang^2^, Yongxin Dai^1^, Xianchong Wan^1,*^, and Shirong Liu^3^

^1^Institute of New Forestry Technology, Chinese Academy of Forestry, Beijing, 100091, China

^2^ College of Forestry, Shanxi Agricultural University, Taigu 030800, China

^3^ Laboratory of Forest Ecology and Environment of State Forestry Administration, Institute of Forest Ecology, Environment and Protection, Chinese Academy of Forestry, Beijing 100091, China

^*^e-mail: [wxc@caf.ac.cn](mailto:wxc@caf.ac.cn)

Hydraulic status, growth and NSC of all saplings were measured in January 2015, which was middle stage of dry season and before Scs renewed its leaves.

Insect outbreaks in March to April

Shoot NSC of defoliated saplings was measured after 20 days of defoliation treatment


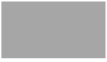


**Dry Season**


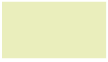


**Wet Season**


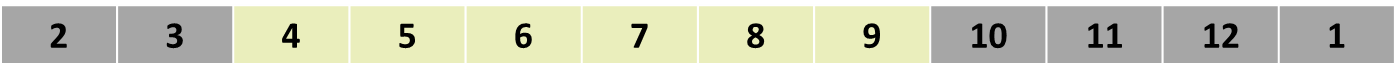


Scs renews its leaves in February

Artifical defoliation treatment was conduced in the end of March

Enr renews its leaves in May

Supplemental Fig 1. The phenology diagram of *Schima superba* (Scs) and *Engelhardia roxburghiana* (Enr)

Supplemental Fig 2

The experiment was conducted in the national reserve, sample sizes for a destructive test are limited by the regulation. For avoiding repeated defoliation of the young trees, we did not harvest all leaves for measuring their area. Instead, we estimated total leaf area by leaf area per branch (measured) multiplied by the number of branches. Thus, the data are submitted as a supplemental material.
